# Deep Sequencing of Small RNAs in Tomato for Virus and Viroid Identification and Strain Differentiation

**DOI:** 10.1371/journal.pone.0037127

**Published:** 2012-05-18

**Authors:** Rugang Li, Shan Gao, Alvaro G. Hernandez, W. Patrick Wechter, Zhangjun Fei, Kai-Shu Ling

**Affiliations:** 1 U.S. Vegetable Laboratory, USDA-Agricultural Research Service, Charleston, South Carolina, United States of America; 2 Boyce Thompson Institute for Plant Research, Cornell University, Ithaca, New York, United States of America; 3 W.M. Keck Center for Comparative and Functional Genomics, University of Illinois at Urbana-Champaign, Urbana, Illinois, United States of America; 4 USDA Robert W. Holley Center for Agriculture and Health, Ithaca, New York, United States of America; Virginia Tech, United States of America

## Abstract

Small RNAs (sRNA), including microRNAs (miRNA) and small interfering RNAs (siRNA), are produced abundantly in plants and animals and function in regulating gene expression or in defense against virus or viroid infection. Analysis of siRNA profiles upon virus infection in plant may allow for virus identification, strain differentiation, and *de novo* assembly of virus genomes. In the present study, four suspected virus-infected tomato samples collected in the U.S. and Mexico were used for sRNA library construction and deep sequencing. Each library generated between 5–7 million sRNA reads, of which more than 90% were from the tomato genome. Upon *in-silico* subtraction of the tomato sRNAs, the remaining highly enriched, virus-like siRNA pools were assembled with or without reference virus or viroid genomes. A complete genome was assembled for *Potato spindle tuber viroid* (PSTVd) using siRNA alone. In addition, a near complete virus genome (98%) also was assembled for *Pepino mosaic virus* (PepMV). A common mixed infection of two strains of PepMV (EU and US1), which shared 82% of genome nucleotide sequence identity, also could be differentially assembled into their respective genomes. Using *de novo* assembly, a novel potyvirus with less than 60% overall genome nucleotide sequence identity to other known viruses was discovered and its full genome sequence obtained. Taken together, these data suggest that the sRNA deep sequencing technology will likely become an efficient and powerful generic tool for virus identification in plants and animals.

## Introduction

Tomato (*Solanum lycopersicum*) is the world’s second most economically important vegetable, after potato, with a total world production in 2009 approaching 153 million tons in 173 countries [1]. The leading tomato producing countries are China, the United States, Italy, and Spain. Although most of the tomatoes are field-grown, protected production systems have increased significantly, particularly in Europe and North America, where several emerging viral diseases have caused serious damages to tomato crops in recent years [2].

In the last decade, *Pepino mosaic virus* (PepMV), genus *Potexvirus* and family *Alphaflexiviridae*, has emerged as one of the most economically important viruses infecting greenhouse tomatoes worldwide [3]. Several distinct genotypes of PepMV have been identified, including EU, US1 and CH2 [4], [5], [6], [7]. In recent years, other emerging diseases that are caused by several pospiviroids have been identified in North America [8], [9], [10], [11], are causing great concern to greenhouse tomato growers. In addition, there is always a concern that other, currently uncharacterized viruses may also be involved in these disease outbreaks.

Timely and accurate detection of phytopathogens is a prerequisite in making effective disease management decisions. Most virus detection methods [e.g., enzyme-linked immunosorbent assay (ELISA), polymerase chain reaction (PCR), or microarray] depend on prior knowledge of antibody or sequence of the potential virus. Advancement in next generation sequencing (NGS) technologies has provided a powerful alternative for total pathogen characterization without *a priori* knowledge.

A metagenomic survey of microbes in honey bee colony collapse syndrome demonstrated the successful use of this approach in identifying a virus as the possible causal agent for this disorder [12]. Using sRNA assembly and analysis, Wu et al. [13] were able to identify several known and unknown viruses infecting invertebrate cell lines. In plant systems, NGS technologies have been applied for virus and viroid identification [14], [15], [16], [17], [18], [19], [20]. Several different RNA template preparations have been attempted for use in NGS, including total plant RNA, double-stranded RNA (dsRNA) and small RNA (sRNA). Deep sequencing of sRNAs has been shown to be the most promising as a detection and identification technique with abilities to identify RNA and DNA viruses in sweet potato [16] and pospiviruses in grapevine [17]. Hagen et al. [19] extended NGS application to identify RNA and DNA viruses in tomato and squash. A new computational algorithm has also been developed to allow for a homology-independent discovery of viroids [21].

sRNAs are produced abundantly in plants and animals and are involved in regulating gene expression. They also have been shown to function in defense against virus infection [22]. RNA silencing (RNAi) constitutes a fundamental antiviral defense mechanism in eukaryotic organisms [23]. Several key steps have been characterized in siRNA biogenesis and targeting of viral RNA for degradation/gene silencing [24]. Plant viral RNAs have been shown to be inducers as well as targets of RNA silencing. Abundant numbers of siRNAs have been shown to accumulate in plants during viral infection [25]. The siRNAs are processed either from dsRNAs or from structured single-stranded RNAs (ssRNAs) by RNase III-like enzymes, such as DICER. In plants, there are at least four classes of DICER-like enzymes (DCL1 to DCL4) [26]. Virus derived siRNAs, in association with Agonautes (AGOs), guide the RNA-induced silencing complex (RISC) to target a viral genome for degradation. DCL4 and DCL2 are two common plant DICERs, generating 21- and 22-nt siRNAs, respectively. DCL3 primarily generate 24-nt siRNA. By sequencing and assembly of siRNAs, a virtual virus genome may be generated [13], [16], [19]. Despite initial successes in virus identification using deep sequencing of siRNAs, a full or near complete virus genome is usually only achieved through assembly of siRNAs onto a closely related reference virus genome [16]. There still remains significant challenge in *de novo* assembly of siRNAs to achieve a complete genome for a novel virus or a new genotype where a reference genome sequence is not available or not reliable. To address these challenges, Isakov et al. [27] described a concept of short RNA subtraction and assembly (SRSA) technology, which can boost pathogen-related siRNA signal through subtraction of host-derived sRNAs, resulting in the identification of the *Human immunodeficiency virus* (HIV) and a mycoplasma in human cells. It is believed that such enrichment of pathogen-related sRNAs will enhance the chance to identify these viruses with very low titer.

We describe here a general bioinformatics pipeline that incorporates an enrichment procedure through *in silico* subtraction of host plant-related sRNAs, followed by Sanger sequencing of RT-PCR products to allow for an efficient generation and validation of complete virus and viroid genome sequences. We were able to effectively identify both PepMV and *Potato spindle tuber viroid* (PSTVd) in a mixed infection and to achieve a strain-specific differentiation of PepMV isolates. In addition, by applying this strategy we were able to identify a novel potyvirus from infected tomatoes, obtaining its complete genome sequence without *a priori* knowledge of its existence.

## Results

### Enrichment of Virus-like sRNAs through Subtraction of Host Plant Genome-specific Sequences

Four virus-infected tomato samples with various disease symptoms, including chlorosis, mosaic stunting, leaf deformation or fruit marbling (Fig. S1), were originally collected from greenhouses in the U.S. or Mexico. PepMV is a chronic disease problem in greenhouse tomatoes in the U.S. [28], thus it was not unexpected that the three U.S. samples (CAHN8, EF09_58 and EF09_60) tested in our study were found to be positive for PepMV. However, the status of other virus or viroid involvement in these U.S. samples was not clear at the time. For the Mexican sample (MX9354), an extensive effort with a panel of virus tests against common greenhouse tomato viruses [2] failed to identify a causal agent to the disease. However, a virus was suspected due to our finding that the infection could be transmitted mechanically to tomato and several other indicator plants including tobacco and eggplant.

**Figure 1:**
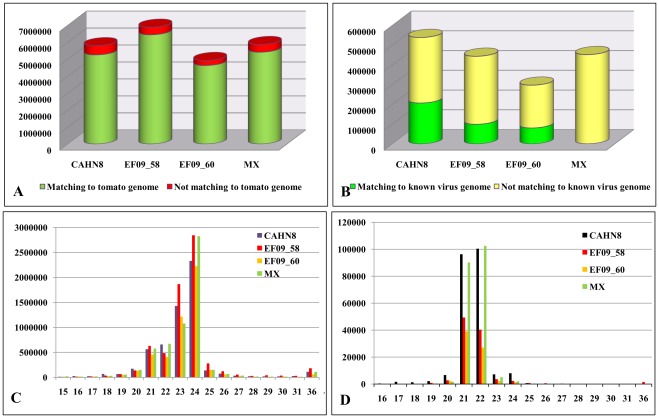
Small RNA (sRNA) profiles in four suspect virus-infected tomato samples. **A**. Enrichment of a virus-like siRNA pool through subtraction of host tomato sRNAs. **B**. Enriched virus-like sRNAs matching known virus and viroid genomes. **C**. Size distribution of total sRNAs (from 15 to 36 nt) in the four samples. **D**. After subtracting host sRNAs, size distribution of siRNAs in each library against respective virus and viroid.

Four individual sRNA libraries were prepared from the purified sRNAs of these tomato samples using unique barcoded-adapters, which were pooled and sequenced on an Illumina Genome Analyzer IIx. Each library generated between 5–7 million reads (Fig. 1A), with an average of 2–3 million unique reads. The majority of those reads were 21–24 nt in length, with a peak at 24 nt for all samples (Fig. 1C).

To enrich each pool of siRNAs originating from viruses, we first aligned the sRNA reads to the tomato genome sequence [29] to remove tomato host-specific sRNAs. We found that more than 90% of sRNAs in each library were actually tomato origin (Fig. 1A). Unmapped sRNA reads, with highly enriched viral siRNAs, were only 6.4 to 10.2% of the total sRNA counts in each library. Analysis of the enriched pool of virus-derived siRNAs through reference-guided assembly to known virus and viroid genomes revealed 22.4–39.4% of siRNAs with virus/viroid origin (Fig. 1B). In comparison, using non-enriched total sRNA pools, only 1.4 to 3.6% would be considered as viral siRNAs in a library. However, sRNAs in the Mexican sample (MX9354) could not be aligned to any known tomato virus or viroid sequence, suggesting an unknown etiology was likely involved. Before subtraction, the majority of sRNAs were associated with the host plant (tomato) and peaked at 24 nt size class (Fig. 1C). After subtraction of the tomato genome-associated sRNAs, virus-specific siRNAs were shown to be predominantly in 21 nt and 22 nt size classes (Fig. 1D).

### Small RNA Assembly and Strain-specific Identification of Viruses and Viroids

Initial sRNA assembly was conducted against known virus and viroid genome databases based on their alignments to these reference genomes. Both (+) and (−) polarity were considered in siRNA assembly to the reference genomes. A gap was considered when a reference genome region was not covered by either polarity of siRNAs.

**Figure 2:**
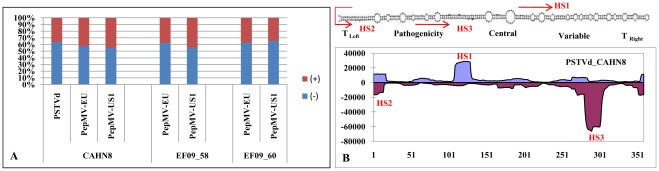
Ratio of virus (+) and (−) polarity siRNAs and hotspots in *Potato spindle tuber viroid* A. Ratio of (+) and (**−**) polarity siRNA reads for PSTVd and both stains of PepMV (EU and US1) in the three U.S. tomato samples. **B**. Distribution of siRNAs along the *Potato spindle tuber viroid* (PSTVd) genome in both (+) and (**−**) polarities.

**Figure 3:**
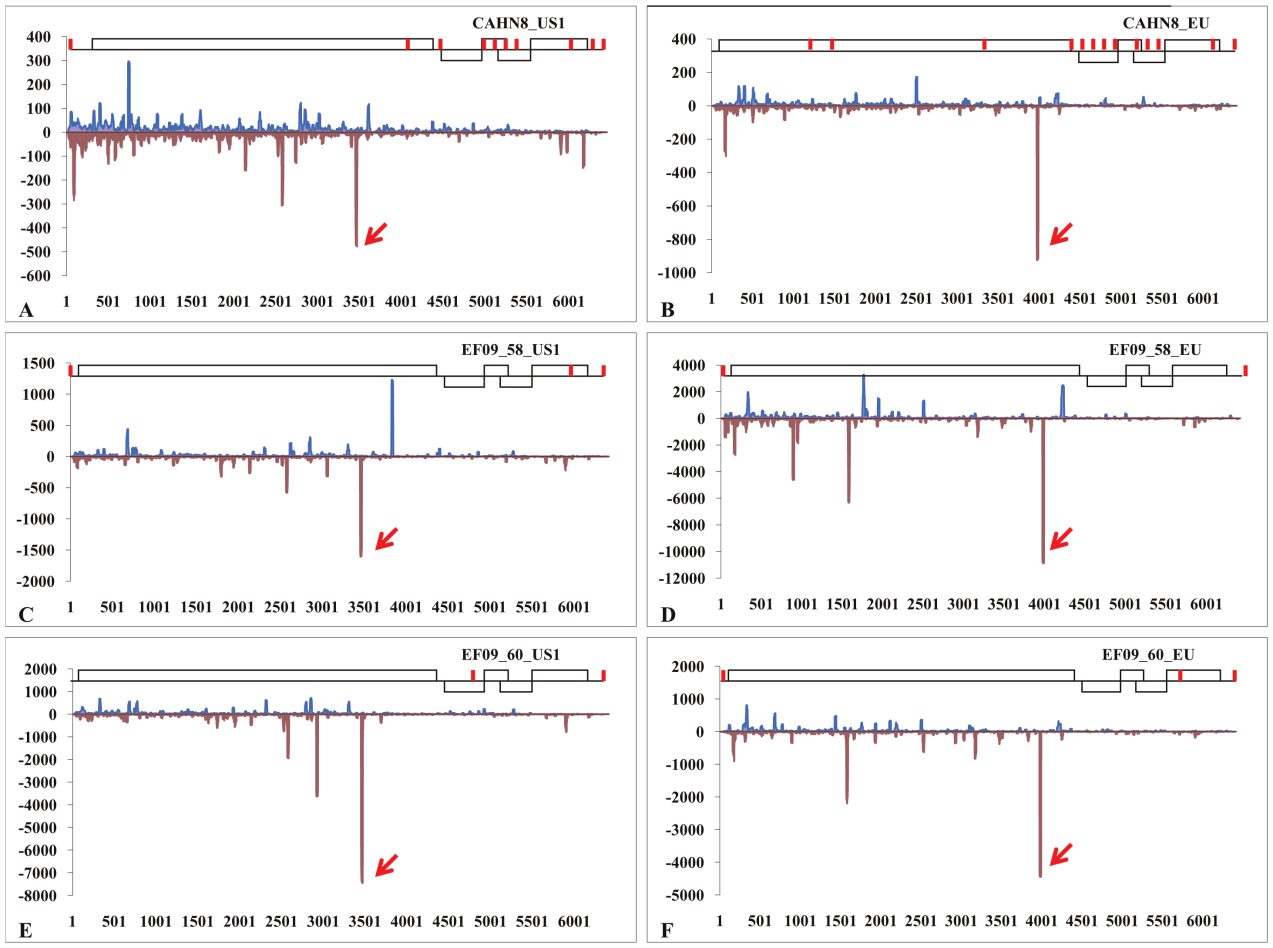
Profiling of siRNAs along the respective strains of *Pepino mosaic virus* in three U.S. tomato samples. CAHN8: top panels **A** and **B**; EF09-58: middle panels **C** and **D**; and EF09-60: bottom panels **E** and **F**. Red lines above the model PepMV genome structures depict broken coverage by either sense or antisense siRNAs. The hot spot for each PepMV isolate is indicated with an arrow.

The siRNAs generated in sample CAHN8 were matched to two PepMV strains (EU and US1, which share only 82% nucleotide sequence identity) and also to PSTVd (Fig. 2A). A complete genome of PSTVd was assembled with siRNAs, without any gaps (Fig. 2B). The siRNA assembled PSTVd sequence was nearly identical (with only a single base insertion) to the reference genome of PSTVd-CA1 (GenBank accession No. HM753555). The genome of PepMV-US1 isolate in CAHN8 was covered by 9 contigs, the longest one was 4,219 nt (Fig. 3A). Each contig was separated by an average of 6 nt. The genome of PepMV-EU in CAHN8 was covered by a total of 13 contigs spanning 98% of the genome leaving small gaps (1–29 nt, with an average of 14 nt) and a missing 3′ terminal sequence. The largest contig generated for this isolate was 2,694 nt (Fig. 3B). The genome sequences assembled in two other U.S. samples (EF09_58 and EF09_60) also contained two strains of PepMV (EU and US1) (Fig. 2A). However their genome sequence assembly was much more complete than that of CAHN8. For PepMV-US1 in EF09_58, two major contigs (6,070 nt and 330 nt) were separated by only a 3 nt gap at position 6077–6079 nt (Fig. 3C). Similarly, a second PepMV genotype (EU) in the EF09-58 sample contained a near-complete virus genome (6,398 nt), lacking sequence data only in the 5′ and 3′ terminal regions (3 nt and 15 nt, respectively) (Fig. 3D). In EF09_60, two major contigs were separated by a 3 nt gap in either genotypes. The two contigs for PepMV-US1 were 4,829 nt and 1,577 nt (Fig. 3E), and the two other contigs for PepMV-EU were 5,568 nt and 820 nt (Fig. 3F).

### Gap Filling and Validation of the Synthetic Genome Sequences by Sanger Sequencing

Validation of the synthetic virus genome upon assembly of the virus-specific siRNA was performed using RT-PCR with overlapping sequence fragments along the identified virus genome. The 5′ terminal sequences were determined using the Rapid Amplification of cDNA Ends (RACE) system (Ambion, Austin, TX). In this experiment, we confirmed the authenticity of two distinct genotypes of PepMV in each of three U.S. samples (CAHN8, EF09_58 and EF09_60), and their complete genome sequences were determined [GenBank Accession No: JQ314457 (PepMV-EU_CAHN8); JQ314458 (PepMV-US1_CAHN8); JQ314459 (PepMV-EU_EF09_58); JQ314460 (PepMV-US1_EF09_58); JQ314461 (PepMV-EU_EF09_60); JQ314462 (PepMV-US1_EF09_60)]. Furthermore, PSTVd genome sequence in CAHN8 (GenBank Accession No. JQ806338) was also validated through Sanger sequencing to be nearly identical to PSTVd-CA1 (GenBank Accession No. HM753555).

**Figure 4:**
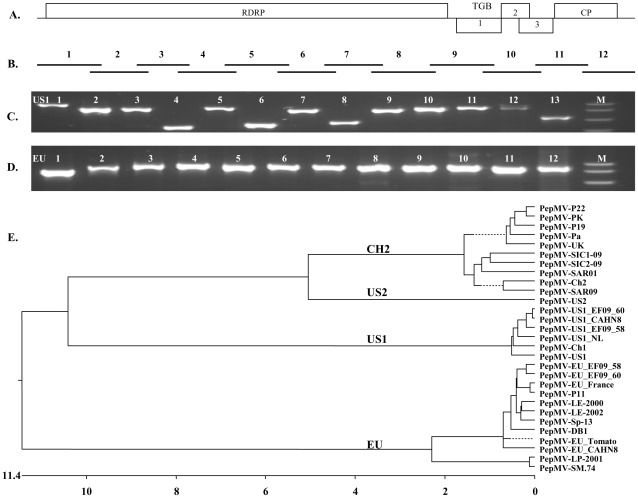
Validation and confirmation of the siRNA derived sequences of two strains of *Pepino mosaic virus* through Sanger sequencing. A. A general PepMV genome organization includes RNA-dependant RNA polymerase (RDRP), triple gene block (TGB) 1, 2 and 3, and coat protein (CP). B. Schematic presentation of step-wise overlapping RT-PCR products generated for sequence confirmation. C. Example of RT-PCR products generated using the US1 strain-specific primers. D. Example of RT-PCR products generated using the EU strain specific primers. M is a DNA ladder with 300, 500, 750 and 1000 bp, respectively. E. Phylogenetic relationship of *Pepino mosaic virus* isolates generated with full genomic sequences showing the newly characterized isolates (EF09-58, EF09-60 and CAHN8) are closely related to the EU or US1 genotypes. The isolates of PepMV and their respective GenBank accession numbers, PepMV-EU_CAHN8: JQ314457; PepMV-US1_CAHN8: JQ314458; PepMV-EU_EF09-58,JQ314459; PepMV-US1_EF09-58: JQ314460; PepMV-EU_EF09-60: JQ314461; PepMV-US1_EF09-60: JQ314462; PepMV-P22: HQ650560; PepMV-PK: EF408821; PepMV-P19: HQ650559; PepMV-Pa: FJ612601; PepMV-UK: FJ212288; PepMV-SIC1-09: HQ663891; PepMV-SIC2-09: HQ663892; PepMV-SAR01: HQ663893; PepMV-Ch2: DQ000985; PepMV-SAR09: HQ663890; PepMV-US2: AY509927; PepMV-US1-NL: FJ940225; PepMV-Ch1: DQ000984; PepMV-US1: AY509926; PepMV-EU-France: AJ438767; PepMV-Sp-13: AF484251; PepMV-LE-2000: AJ606359; PepMV-LE-2002: AJ606360; PepMV-P11: JN133846; PepMV-EU-Tomato: FJ940223; PepMV-DB1: FJ940224; PepMV-LP-2001: AJ606361, and PepMV-SM.74: AM109896.

To validate a siRNA-assembled virus genome, we designed a step-wise primer walking system (Fig. 4B) to obtain RT-PCR products with overlapping viral genome sequence (Fig. 4C, 4D). To facilitate direct sequencing of RT-PCR products, the virus-specific primers were modified to contain M13 Forward and Reverse primer sequences in each pair (Table S1). The sequences generated between the siRNA assembly and Sanger sequencing were essentially identical, thus supporting the reliability of deep sequencing of sRNA technology for virus and viroid identification and discovery. Only sequences validated by Sanger sequencing were deposited into the GenBank.

A mixed infection of two genotypes of PepMV was found in all three U.S. greenhouse tomato samples. Upon gap filling and 5′ RACE, six complete PepMV genomes, including both EU and US1, were obtained. These PepMV-EU and PepMV-US1 isolates, originating from the same greenhouse in Arizona (EF09_58 and EF09_60), were nearly identical, sharing 99.6% nucleotide sequence identity. On the other hand, PepMV isolates from the California sample (CAHN8) could be differentiated from the Arizona isolates, with only 98.6% nt identity to their respective genotypes (EU and US1). Phylogenetic relationships of these six new PepMV isolates along with 22 previously published PepMV genome sequences were analyzed, and their genotype assignments were found to be in agreement with convention (Fig. 4E). In addition to PepMV isolates, sample CAHN8 also carried a PSTVd sequence. However, no other virus-like sequences could be clearly observed when *de novo* assembled contigs were used to BLAST against the nt and nr databases.

### Identification and Examination of Hot Spot siRNA Sequences

In agreement with a previous observation on siRNA profiles characterized on two pospiviroids in grapevine [17], there are more siRNAs in (**−**) polarity than those in (+) polarity for PSTVd in tomato sample CAHN8. In fact, nearly two thirds (63.6%) of PSTVd siRNAs were in (**−**) polarity, with only one third (36.4%) in (+) polarity (Fig. 2 A). Interestingly, in the case of PepMV, the (**−**) polarity siRNAs were also prevalent over the (+) polarity (Fig. 2 A).

Frequency and distribution of siRNA coverage over the reference virus genomes was not even. Examination of the siRNA profiles revealed three notable hotspots for PSTVd-specific siRNAs. The first hotspot (HS1) was located in the Central to the Variable domains of a typical pospiviroid. The second hotspot (HS2) covered the Terminal Left region in both (+) and (**−**) polarities. The third hotspot (HS3) mapped to the Pathogenicity domain of (**−**) polarity siRNAs (Fig. 2B). The most conserved siRNA sequences for these hotspots are listed in Table S2.

Examination of siRNA profiles for PepMV isolates revealed one major hotspot which was genotype-specific across the three tomato samples examined (Fig. 3). These hotspots were mapped to the (**−**) polarity of the respective genome and were similar in terms of sequence and genome location among various isolates in the same genotype. The most conserved siRNA for the EU genotype (TGACTGTAGAATCAAGATGGT) was located on nt 3990-3970 (Table S3). The most conserved siRNA for the US1 genotype (CAGGAGATTGTCGACTAGCGGC) was mapped to nt 3473-3452 (Table S4). Such genotype specificity may be useful to differentiate one genotype (EU) from the other (US1).

### Identification of a Novel Potyvirus in the Mexican Sample

**Figure 5:**
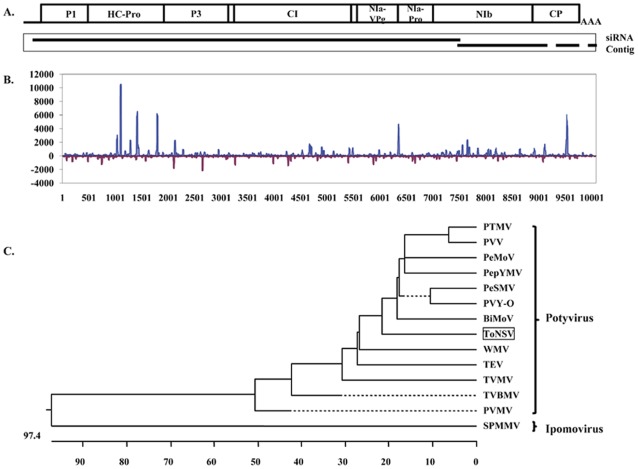
Identification of Tomato necrotic stunt virus (ToNSV) in the Mexican sample (MX) and its relationship with other viruses in the family of Potyviridae. A. Relative placement of *de novo* assembled siRNA contigs onto a general potyvirus genome. B. Distribution of siRNAs along the ToNSV genome in both (+) and (−) polarities. C. Phylogenetic relationship of the newly identified ToNSV genome in relationship to other viruses in the family of Potyviridae. The full name of potyviruses and their respective GenBank accession numbers are: ToNSV-MX9354: JQ314463; PTMV, *Peru tomato mosaic virus*: AJ516010; PVY-O, *Potato virus Y*: HQ912914; PeSMV, *Pepper severe mosaic virus*: AM181350; PeMoV, *Pepper mottle virus*: EU586135; PVV, Potato virus V: NC_004010; PepYMV, *Pepper yellow mosaic virus*: NC_014327; BiMoV, *Bidens mottle virus*: EU250214; WMV, *Watermelon mosaic virus*: DQ399708; PVMV, *Pepper veinal mottle virus*: FM202327; TVBMV, *Tobacco vein-banding mosaic virus*: EF219408; TVMV, *Tobacco vein mottling virus*: X04083; and TEV, *Tobacco etch virus*: EF470242. SPMMV, *Sweet potato mild mottle virus*: NC_003739, genus *Ipomovirus*, was used as an outgroup sequence to anchor the phylogenetic tree.

Through mechanical inoculation, the Mexican sample (MX9354) could induce plant stunting, necrosis, and leaf and fruit deformation symptoms on the inoculated tomato plants (Fig. S1B). However, its causal agent was unknown at the time. *De novo* assembly of virus enriched sRNAs (after subtraction of host-derived sRNAs) generated 54 sequence contigs with lengths ranging from 37 to 8,432 nt. A BLASTX search revealed the two longest contigs (8,432 and 1,146 nt, respectively) had significant alignments (amino acid sequence identity of ∼60%) to polyproteins of several known potyviruses. Closer examination indicated that these two contigs had a 13-nt overlap at ends thus could potentially be joined into one contig (Fig. 5A). To obtain a complete virus genome and to validate the authenticity of this novel virus, overlapping RT-PCR products were produced using primers derived from the two contigs. In general, the sequences obtained from Sanger sequencing were in agreement with those generated from the siRNA assembly, with only three nucleotide differences (Table S6). After completion of the 5′ and 3′ terminal sequencing, the final complete and validated virus genome for the newly identified virus was determined to comprise 10,057 nt excluding the 3′-terminal poly(A) tail (GenBank Accession No. JQ314463). The genome encodes a predicted single polyprotein of 3,132 amino acids with an apparent molecular mass of 354 kDa arranged in a typical potyvirus genome organization (Fig. 5). Based on the severity of symptoms on tomato, we provisionally named this virus “Tomato necrotic stunt virus” (ToNSV). Alignment of the siRNA sequences back to the newly generated ToNSV genome sequence resulted in a fully covered siRNA profile along the genome (Fig. 5B). Phylogenetic analysis clearly placed ToNSV alongside other members of the potyviruses (Fig. 5C). It is worth noting that two additional *de novo* assembled contigs with length of 307 and 79 nt [69 nt after removing the poly(A) tail] can be perfectly aligned to the 3′ end of the ToNSV genome (Fig. 5A and Table S6). These two contigs initially were not identified as virus sequences due to their lack of homology to known virus sequences. Taken together, the four *de novo* assembled contigs covered 98.9% (9,943/10,057) of the ToNSV genome.

### Improvement of *de novo* Virus sRNA Assembly through Subtraction of Host-derived sRNAs

It has been suggested that subtraction of host-derived sRNAs can improve the efficiency of virus sequence identification from short sRNA sequences [27]. In the present study, we compared the assemblies derived from sRNAs of the Mexican sample with and without subtraction of host-derived sRNAs. The assembly with host sRNA subtracted generated 54 contigs ranging from 37 to 8,432 nt with an average length of 240 nt, while the assembly of the complete set of sRNAs generate 1,626 contigs ranging from 30 to 1,192 nt with an average length of 70 nt. Alignments of these contigs to the ToNSV genome clearly showed the advantage of the assembly with host sRNA subtraction, which produced much longer and more completed virus sequences (Table S6). Comparisons of *de novo* assemblies with and without host sRNA subtraction using datasets from the other three samples provided the similar results (data not shown).

## Discussion

Here, we describe a bioinformatics pipeline to efficiently identify tomato viruses and viroids in a mixed infection and to achieve a strain-specific differentiation. This bioinformatics pipeline relied on the use of computer-assisted subtraction of host plant-associated sRNAs. In our experiments, nearly 90% of the total sRNAs from the tomato genome were removed. Highly enriched exogenous siRNAs were used for the assembly of the virus genome, either reference-guided or *de novo*. Complete genomes of six PepMV isolates and a PSTVd isolate were obtained and validated through RT-PCR and Sanger sequencing. Although a similar approach has been used to identify HIV and a mycoplasma in a human cell culture [27], we were able to streamline the process to obtain, for the first time, a complete viral or viroid genome assembly. The precision of the technology was demonstrated to permit a strain-specific differentiation in a mixed infection of two closely related genotypes of an endemicvirus which share 82% nucleotide sequence identity. The advantage of using the subtraction of host associated sRNAs is that it can significantly enhance the ratio of virus-related siRNAs from an average of 2.5% to nearly 40% thus increase the efficiency of *de novo* assembly of virus sequences. The strategy of subtraction of host sRNAs for enrichment of virus-derived siRNAs has not been used in previous studies in plant virus identification [13], [16], [19]. Recently, a fully annotated tomato genome became available [29] and thus provided advantage to the present research. As more and more plant genomes are being sequenced, there is great opportunity in using this subtraction strategy to improve chances of identifying viral siRNAs from mixed host-pathogen samples. In addition, this strategy allows reduction in the number of gaps generated, aiding in assembly of full virus genomes. Although Hagen et al. [19] did not seem to observe significant improvement in the number of larger sized contigs, in the present study we did find the significant improvement in *de novo* assembly of virus sequences by subtraction of host-derived sRNAs. In addition, Hagen et al. [19] pointed out that subtraction of host sRNA would also be beneficial in identifying different viruses in a mixed infection or two closely related strains of a virus in field-collected or pooled samples. Although deep sequencing and assembly of the sRNAs appears to have provided a complete survey of viruses and viroids in these samples, a totally novel virus or viroid that has little homology in nucleotide or amino acid sequence with the known viruses in the current GenBank might have been missed using BLAST. Analysis of sRNA libraries using the newly developed homology-independent algorithm for viroid discovery (21) may help to reach such conclusion.

Analysis of the accumulation of siRNAs has demonstrated that the most prevalent siRNAs were in the (**−**) polarity for both PSTVd and PepMV in tomato. Such preferential accumulation of the (**−**) polarity siRNAs for PSTVd is in contrast with two previous studies, using Sanger sequencing of sRNAs in tomato, in which it was reported that (+) polarity siRNAs in PSTVd [30] and CEVd [31] are more abundant. However, our results appear to be in agreement with a previous effort in deep sequencing of siRNAs for two other pospiviroids (HSVd and GYSVd1) in grapevine [17]. Although the mechanism of biogenesis and accumulation of siRNAs for viroids is still unknown, a general non-symmetric positioning of the two major hotspots (HS1 and HS3) in two opposite polarities suggests a more complex mechanism may be involved. The two prominent peaks of 21- and 22- nt size sRNAs were in agreement with three previous studies [30], [31], [32]. The significant level of PSTVd siRNA accumulation in CAHN8 is intriguing as this seems to have little effect on the severe stunting and chlorosis symptoms present in viroid infected tomato plants. However, significance of the hotspots in affecting viroid infectivity or in interfering with host gene expression is in need of further investigation.

In the case of PepMV, the (**−**) polarity siRNAs were also prevalent over that of the (+) polarity. Previous observation in invertebrate viruses showed that both polarities of siRNAs were present in an equal number [13]. However, Kreuze et al. [16] reported that more positive strand over the negative strand siRNAs was observed in a sweetpotato virus study. The major hotspot (**−**) polarity siRNAs, although located in two different genomic regions of the virus in each specific strain, were all targeting the RNA-dependant RNA polymerase gene, suggesting a potential role in host defense against virus replication. Previous study [24] has shown that there are three classes of siRNAs involved in siRNA biogenesis. The first class of siRNAs is derived from processing the dsRNA during virus replication. The second class of siRNAs is produced by transitive silencing, in which dsRNA is produced from ssRNA templates by cellular RNA-dependent RNA polymerase activity. The third class of siRNAs is the structure-associated siRNAs generated through imperfectly base-paired intramolecular hairpins in viral genomes or transcripts. Currently, we do not know whether the differential siRNA distribution within the hotspots has any biological significance, although the highly coincidental profiles in three independent sequencing experiments are intriguing. In a Drosophila study, Kawamura et al. [33] demonstrated the strand-biased siRNA hot spot could be generated from the stem-loop generating region. The resulting hot spot siRNAs then guide AGO2 to cleave complementary RNA templates. In the case of PSTVd, the hot spot siRNAs in either 21 or 22 nt were in either sense or antisense to the viroid genome (Table S2). For either genotype of PepMV, the hot spot siRNAs in both 21 and 22 nt were in antisense, complementary to the virus genome (Tables S3 and S4). Although PSTVd contains strong stem-loop secondary structure, no obvious stem-loop structure was observed in PepMV genomic regions flanking the primary hot spot in either genotype (using sequence fragment 50 nt in either direction surrounding the hot spot) using RNAfold program (http://rna.tbi.univie.ac.at/cgi-bin/RNAfold.cgi). A mechanism for the possible involvement of the hot spot siRNA in RNA silencing for plant recovery is awaiting further investigation. A fundamental understanding of the mechanism of virus-host interactions may lead us to better use the hotspot siRNA information to manage viral disease infection.

In addition to the major hotspot on the PepMV genome, there were numerous minor hotspots (Fig. 3). Although most of the siRNA classes were 21 or 22 nt (Fig. 1D), we also identified a series of unusual minor hot spot siRNAs of 26–36 nt in association with PepMV-EU in sample EF09_58 (Table S5) which may have some similarity to the Piwi-interacting RNA (28–30 nt) [34]. Alternatively, these unusually long sRNAs may be processed from a DICER-independent biogenesis pathway and AGO2-mediated cleavage of pre-miRNAs, thus generating functional miRNAs independent of DICER [35]. The function of such long sRNAs is still unknown. Nevertheless, it represents a different class of sRNAs in plants during virus infection.

Gap filling and validation of an assembled virus genome from siRNAs was necessary to obtain complete, authentic virus genome sequences. Although assembly of sRNA sequences has been successfully used by a number of researchers to generate virus genome contigs, *de novo* assembly was rarely able to produce a full virus genome [13], [16], [19], [27], [36]. Small gaps between contigs are common even after being ordered on a reference virus genome. To achieve an artificial full genome, Hagen et al. [19] generated a synthetic full-length template by artificially filling gaps using the reference genome sequence of the closest virus. Unfortunately, these synthetic virus genomes can undermine the use of NGS sequencing and *de novo* assembly technologies, and can potentially cause confusion in the literature. Therefore, in the present study, we took extra steps, not only in filling the gaps but also in validating the siRNA assembled virus genome sequences using Sanger sequencing of the overlapping RT-PCR products. This strategy enabled us to confirm the presence of a mixed infection of two closely related genotypes of PepMV (overall 82% sequence identity), which also allowed us to obtain a complete virus genome for a novel virus without *a priori* knowledge. We believe that these confirmation procedures are necessary in order to generate a complete and correct genomic sequence when using the current *de novo* assembly strategy. Even though a synthetic viral genome may serve as a reference sequence for the discovery of a very closely related genotype of a target virus, authenticity of these siRNA generated genomic sequences need to be validated by Sanger sequencing of the RT-PCR products.

Identification of a novel potyvirus (ToNSV) without *a priori* knowledge, and associated confirmation of a complete virus genome by Sanger sequencing clearly demonstrates the power and efficiency of our bioinformatics pipeline. ToNSV is causing serious damage in certain Mexican tomato crop production systems. Characterization of ToNSV’s etiology has been hampered by the novelty of this new virus, which has less than 60% nucleotide sequence identity to other known viruses. Availability of the complete viral genome sequence will permit us to further characterize its biological properties, to develop serological and molecular detection technologies, and to better understand the factors influencing disease epidemiology. With this knowledge, we can better devise an effective disease management strategy.

Even with the rapid advances in sequencing technologies and decrease in cost of sequencing, major challenges still remain in the development of an efficient bioinformatics pipeline for effective virus identification and complete genome assembly and validation. As more plant and animal genome sequences become available, this subtraction and assembly strategy should avail itself to broader applications in timely and thorough detection of existing, emerging, or novel viruses and viroids.

## Materials and Methods

### Virus Sources and Total RNA Preparation

Four tomato samples with suspected virus infection were originally collected from California (CAHN8), Arizona (EF09_58 and EF09_60), and Mexico (MX9354) and maintained on tomato (cv. Moneymaker) through mechanical inoculation in a greenhouse. Total plant RNA was purified from 3 g of leaf tissue using the TRIzol method following manufacturer’s instructions (Invitrogen, Carlsbad, CA). The purified RNA preparations were evaluated on a Bioanalyzer (Agilent Technology, Santa Clara, CA) and shipped in ethanol solution to the University of Illinois Genomic Center for small RNA library construction and sequencing.

### Small RNA Library Construction and Illumina Sequencing

Small RNA libraries were constructed from 1.0 µg of total RNA using the “Small RNA v1.5 Sample Prep” kit from Illumina (San Diego, CA). Unique 4 bp barcode sequences were added to the 5′ adaptors so that the libraries could be multiplexed and the reads sorted by samples. The barcoded libraries were pooled in equimolar concentration and sequenced on a single lane on a Genome Analyzer IIx for 41 cycles and the files were processed with Illumina’s CASAVA pipeline (version 1.5).

### Small RNA Sequence Processing, Assembly and Virus Genome Identification

sRNA sequence processing, assembly, and virus and viroid genome identification were conducted using a custom bioinformatics pipeline. Briefly, raw Illumina sRNA reads were first processed to trim adaptor and barcode sequences, and then assigned to individual samples based on the barcode sequences they contained. Trimmed sRNA sequences shorter than 15 nt were discarded. Subtraction of sRNAs derived from the host genome was performed by aligning sRNAs to the tomato genome sequences using the BWA program [37] with the edit distance set to 1. The resulting highly enriched exogenous siRNAs from each sample were aligned to the known virus and viroid genomes collected from the NCBI GenBank database, using BWA with the edit distance of 1. The synthetic contigs were derived based on the alignment of sRNAs to known virus and viroid genomes. sRNAs from each sample were also assembled *de novo* using Velvet [38]. Following assembly, sRNAs were aligned back to the assembled contigs using BWA, and the base coverage at each position of the contigs was derived based on the mpileup file generated from the alignments using SAMtools [39] and used to correct potential base calling errors in the *de novo* assemblies by replacing with the most frequent base covering that specific position. The resulting final contigs were used to query against the GenBank nt and nr databases, respectively, using the BLAST program [40]. Contigs with significant similarity to known virus and viroid sequences in the databases were identified as candidate virus or viroid genome sequences.

### Validation and Completion of Viral Genome Sequences with Sanger Sequencing

In an effort to generate complete virus genome sequences, specific primers were designed to fill the gaps in the assemblies and the 5′ terminal sequence was obtained through rapid amplification of cDNA ends (RACE). The authenticity of a synthetic siRNA assembled virus genome sequences was validated through Sanger sequencing of the RT-PCR products generated with primers derived from the reference genome sequences for PepMV-US1 (Genbank Accession No. DQ000984) or PepMV-EU (GenBank Accession No. AJ438767). RT-PCR products were directly sequenced using the common sequencing adaptors (M13 Forward and Reverse primers) linked to each virus specific primer (Table S1). In some cases, when direct sequencing did not yield high quality sequence data, the RT-PCR product was cloned and sequenced. For some genomic regions, where predicted sizes of RT-PCR were not amplified in initial experiments, new virus-specific primers were designed based on the flanking virus-specific siRNA sequences.

To determine the 5′terminal sequence for PepMV isolates, the First Choice RLM-RACE kit (Ambion, Austin, TX) was used. For the US1 isolates: an outer primer (KL11-78, nt positions 732-713: 5′-GTGAAAGTATGCCCCACCAC) and another inner primer (KL11-77, nt positions 235-216: 5′-GGGTGTCGGCTTCATAATCA) were used for the first and second round of amplification of the virus specific cDNA. PCR products with expected sizes were cloned using TOPO TA cloning kit (Invitrogen, Carlsbad, CA) and selected clones (4–6 clones) sequenced. Similarly for EU isolates, an outside primer (KL11-105, nt positions 734-715: 5′-GTGGAAATATGCCCCACCAC) and an inner primer (KL11-104, nt positions 243-224: 5′-TCTCAAGGGTGTCTGCTTCA) were included in the reactions.

For the novel virus (ToNSV) without a reference sequence, primers were designed based on the two assembled siRNA contigs. RT-PCR was used to validate the assembled contigs with overlapping RT-PCR products (Table S1). To determine the exact 5′ terminal sequence of the new virus, a different RACE system was employed. Because of the predicted VPg-linked viral RNA for a potyvirus, the 5′terminal sequence of the new virus was determined with an Invitrogen 5′RACE kit using viral RNA extracted from a partial purified virus preparation through polyethylene glycol (PEG) precipitation [41]. After cDNA synthesis using random primer, the 3′ end of cDNA was tailed with dCTP. First round of PCR amplification was conducted using the abridged anchor primer and a virus-specific outer primer (KL11-249, nt positions 509-490: 5′-GCTGCTTACGCACCCTACTC). To obtain 5′RACE products, two independent experiments were performed in nested PCR using abridged universal amplification primer in combination with two individual virus-specific inner primers (KL11-189, nt positions 227-208: 5′-CAAAACACATTGCCATTGGA and KL11-250, nt positions 361-342: 5′-CACCACGGAAGTTGTCACAG).

Because both PepMV (a potexvirus) and ToNSV (a potyvirus) were expected to contain poly(A) tails at their 3′ termini, the 3′ terminal sequence was determined by sequencing the cloned RT-PCR products generated using an oligo(dT) primer (5′-GGTCTCGAGTTTTTTTTTTTTTTT-3′) and their respective virus-specific forward primers (PepMV-US1: 5′-AAAGTTCAAAATCCGCACAA-3′; PepMV-EU: 5′-GCTGCTCACTCCGTAGC-3′, and TNSV: 5′-ACTCGAGCCACAATGCAAC-3′).

DNASTAR (Lasergene 8) was used to assemble and analyze each genomic sequence. A BLAST analysis of the complete genomic sequence and deduced polyprotein sequence was performed to identify closely related viral sequences available in the GenBank. The pairwise sequence comparison (PASC) program [42] was used to classify the newly identified virus by pairwise global alignment of selected viruses in the family Potyviridae. A multiple sequence alignment of complete genomes was performed with Clustal W [43] and its phylogenetic tree generated with 1,000 bootstraps.

## Supporting Information

Figure S1 **The four tomato samples exhibiting virus-like symptoms were originally collected from greenhouse grown tomato plants in the U.S. or Mexico.** A). CAHN8, with chlorosis and mosaic, in southern California. B). MX 9354, stunting, necrotic spot and leaf and fruit deformation, near Mexico City. C). EF09-58, mosaic and purpling leaf, in Arizona. D). EF09-60, marbling fruit, in Arizona.
(TIF)

Table S1 **Oligonucleotide primers used in generating RT-PCR products for Sanger sequencing to obtain full genomic sequences for the two genotypes of** ***Pepino mosaic virus*** **(EU and US1) and for the new potyvirus (Tomato necrotic stunt virus).**
(DOC)

Table S2 **siRNA hot spot sequences and relative positions on** ***Potato spindle tuber viroid*** **in isolate CAHN8.**
(DOC)

Table S3 **Conserved siRNAs on the hotspots against** ***Pepino mosaic virus*** **EU strain in the three tomato samples.**
(DOC)

Table S4 **Conserved siRNA on the hotspots against** ***Pepino mosaic virus*** **US1 strain in the three independent samples.**
(DOC)

Table S5 **An unusual hot spot with siRNAs of upto 36 nt to PepMV-EU in the isolate EF09-58.**
(DOC)

Table S6 **Alignments of** ***de novo*** **assembled contigs to the ToNSV genome.**
(XLSX)
