# Supplementary figures and images for: Deep Sequencing of Small RNAs in Tomato for Virus and Viroid Identification and Strain Differentiation
Source: PLoS One. 2012 May 18;7(5):e37127. doi: 10.1371/journal.pone.0037127 (PMC3356388; doi:10.1371/journal.pone.0037127)

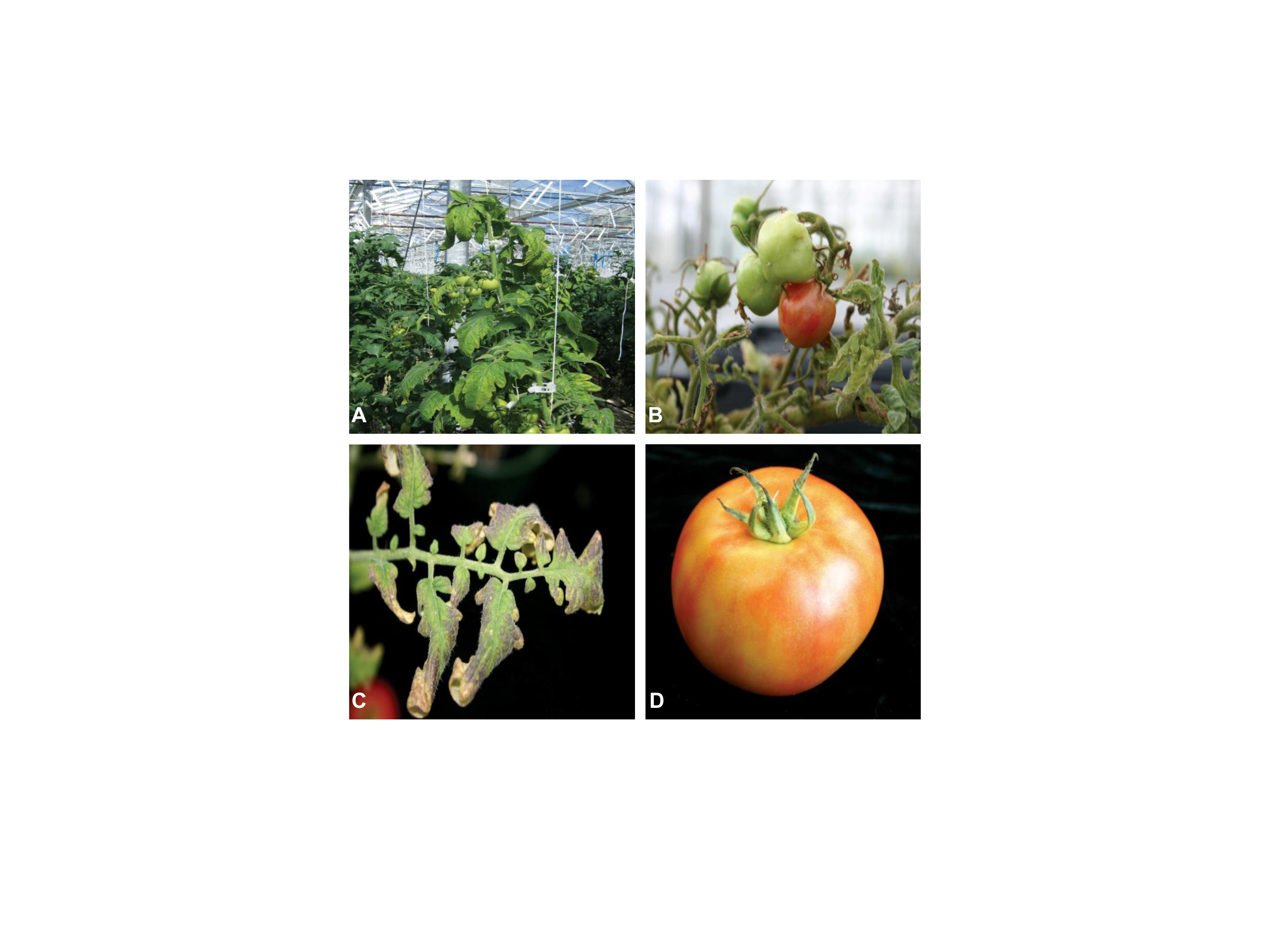

Supplement: Figure S1 — The four tomato samples exhibiting virus-like symptoms were originally collected from greenhouse grown tomato plants in the U.S. or Mexico. A). CAHN8, with chlorosis and mosaic, in southern California. B). MX 9354, stunting, necrotic spot and leaf and fruit deformation, near Mexico City. C). EF09-58, mosaic and purpling leaf, in Arizona. D). EF09-60, marbling fruit, in Arizona. (TIF) [file pone.0037127.s001.tif]
