# Supplementary material for: Deep Sequencing of Small RNAs in Tomato for Virus and Viroid Identification and Strain Differentiation
Source: PLoS One. 2012 May 18;7(5):e37127. doi: 10.1371/journal.pone.0037127 (PMC3356388; doi:10.1371/journal.pone.0037127)
Supplement: Table S1 — Oligonucleotide primers used in generating RT-PCR products for Sanger sequencing to obtain full genomic sequences for the two genotypes of Pepino mosaic virus (EU and US1) and for the new potyvirus (Tomato necrotic stunt virus). (DOC) [file pone.0037127.s002.doc]

Table S1. Oligonucleotide primers used to regenerate overlapping RT-PCR products along the respective virus genome.

| **Fragment** | **Forward Primer (5'-3')*** | **Reverse Primer (5'-3')** | **PCR Size (bp)** | **Genome Region** |
| --- | --- | --- | --- | --- |
|  | **Pepino mosaic virus US1 genotype (PepMV-US1)** |  |  |  |
| 1 | KL11-64 (CAGGAAACAGCTATGACGAAAACAAAACATAACCAAAAC) | KL11-163 (TGTAAAACGACGGCCAGTATTGGATTGCCCTGGAGCGT) | 988 | 1-988 |
| **2** | KL11-65 (CAGGAAACAGCTATGACTGCGTTACATGAGAAGAGCTG) | KL11-79 (TGTAAAACGACGGCCAGTTCAATCATGCACCTCCAGTC) | 832 | 389-1229 |
| **3** | KL11-66 (CAGGAAACAGCTATGACCGCCCTTATATAGGACCTTTCA) | KL11-80 (TGTAAAACGACGGCCAGTCAGATGCATAACTAGCCCATGT) | 852 | 878-1729 |
| **4** | KL11-164 (CAGGAAACAGCTATGACGCTGACAACTTCGCTGTTATAAATG) | KL11-165 (TGTAAAACGACGGCCAGTTGATGTCCTGAATTGGTTGGATTA) | 349 | 1621-1969 |
| **5** | KL11-68 (CAGGAAACAGCTATGACCTTCTCAATGCCCATGGTTT) | KL11-82 (TGTAAAACGACGGCCAGTAGGGCACCAAGATGGGTACT) | 844 | 1888-2731 |
| **6** | KL11-166 (CAGGAAACAGCTATGACTGTGGTTTCTATTTGAACGCTACG) | KL11-167 (TGTAAAACGACGGCCAGTATCTCAGTCTTTTCATCCCGGTAT) | 419 | 2587-3005 |
| **7** | KL11-70 (CAGGAAACAGCTATGACTCAATACTGGCCCCAACAAT) | KL11-84 (TGTAAAACGACGGCCAGTTTCCAACCATTGAGAACAAAA) | 794 | 2906-3699 |
| **8** | KL11-71 (CAGGAAACAGCTATGACTGTGCAGCTGAAGTCAAGAA) | KL11-49 (5’TTTAGCGTTAAGCTTAATGTTGA) | 475 | 3394-3870 |
| **9** | KL11-168 (CAGGAAACAGCTATGACCAATCCCAAGATGGAGCTATGTTA) | KL11-169 (TGTAAAACGACGGCCAGTTCGGGTAAGCGGAAAATAAAGTT) | 808 | 3763-4570 |
| **10** | KL11-73 (CAGGAAACAGCTATGACCCAAAAGCAACACATTGACG) | KL11-87 (TGTAAAACGACGGCCAGTCAGCAACAGGTTGGTAATGG) | 840 | 4387-5228 |
| **11** | KL11-74 (CAGGAAACAGCTATGACTGAATTCTTTGACCCATTTCAA) | KL11-88 (TGTAAAACGACGGCCAGTAAATCACTGAGGCTAGGAGCTG) | 876 | 4875-5750 |
| **12** | KL11-75 (CAGGAAACAGCTATGACAGCCGTACTACTCACCAGCA) | KL11-89 (TGTAAAACGACGGCCAGTGGAAATTTTCTGCCTGGCTA) | 845 | 5405-6249 |
| **13** | KL11-76 (CAGGAAACAGCTATGACATCTAGCGCGTGCTTATGCT) | KL11-90 (TGTAAAACGACGGCCAGTAAATTACAAAAGCAATTTATTG) | 542 | 5873-6414 |
|  |  |  |  |  |
|  | **Pepino mosaic virus EU genotype (PepMV-EU)** |  |  |  |
| **1** | KL11-54 (GAAAACAAAACAAATAAACAAAT) | KL11-105 (TGTAAAACGACGGCCAGTGTGGAAATATGCCCCACCAC) | 737 | 1-737 |
| **2** | KL11-92 (CAGGAAACAGCTATGACTGAGAAGAGCTGCTGTGCAT) | KL11-106 (TGTAAAACGACGGCCAGTTCAATCATGCACCTCCAGTC) | 832 | 403-1234 |
| **3** | KL11-93 (CAGGAAACAGCTATGACGAGACGCCGCTATACAGGAC) | KL11-107 (TGTAAAACGACGGCCAGTGCATAACTGGCCCATGTCTT) | 851 | 879-1729 |
| **4** | KL11-94 (CAGGAAACAGCTATGACAAGAAAGAAAGCAGCTGACTCAA) | KL11-108 (TGTAAAACGACGGCCAGTTGCTTGTGATTTTCCAGAGC) | 869 | 1381-2249 |
| **5** | KL11-95 (CAGGAAACAGCTATGACGGCTTCAAGGGAGACCAACT) | KL11-109 (TGTAAAACGACGGCCAGTTTGGCACTTTGCACTTTTGT) | 817 | 1908-2724 |
| **6** | KL11-96 (CAGGAAACAGCTATGACAAAGCAATGATTCAACCAGGA) | KL11-110 (TGTAAAACGACGGCCAGTTCTTTGGCTTGTTGATGCTG) | 842 | 2382-3223 |
| **7** | KL11-97 (CAGGAAACAGCTATGACTGGATTCCATCCACTTTATCA) | KL11-111 (TGTAAAACGACGGCCAGTTCATTTGTCTGAGCTGTTCTGTT) | 859 | 2893-3751 |
| **8** | KL11-98 (CAGGAAACAGCTATGACTGGGAAATATGTGCTGCTGA) | KL11-112 (TGTAAAACGACGGCCAGTTAAATTGCCGATTGCCTCTT) | 834 | 3390-4223 |
| **9** | KL11-99 (CAGGAAACAGCTATGACCCTTGGCACATTGTCCATTA) | KL11-113 (TGTAAAACGACGGCCAGTGCCAATTTTGGTTCTGGAAA) | 839 | 3881-4721 |
| **10** | KL11-100 (CAGGAAACAGCTATGACCCCTCCAAAAGCAACACATT) | KL11-114 (TGTAAAACGACGGCCAGTCAAACGGTGCAAGTTGTCTC) | 865 | 4388-5254 |
| **11** | KL11-101 (CAGGAAACAGCTATGACAAGTTGACCCCACTGAGCAT) | KL11-115 (TGTAAAACGACGGCCAGTGGGATTTGAGAAGTCAGAAGGA) | 828 | 4901-5728 |
| **12** | KL12-85 (CAGGAAACAGCTATGACGTTTTCCTAAATTTGAAAAT) | KL12-86 (TGTAAAACGACGGCCAGTATTTCAAAGAAATAATTAGG) | 837 | 5575-6413 |
|  |  |  |  |  |
|  | **Tomato necrotic stunt virus (TNSV)** |  |  |  |
| **1** | KL11-172 (CAGGAAACAGCTATGACCTAAATTAAAACATCTCAACACAACTT) | KL11-160 (ATCAGTTTGCGCCTTCAAGT) | 1243 | 1-1243 |
| **2** | KL11-185 (CAGGAAACAGCTATGACAATGCACTCACTAGGGTGAC) | KL11-178 (TGTAAAACGACGGCCAGTTCCCCTTCCAAAGAATTGGT) | 477 | 1127-1603 |
| **3** | KL11-173 (CAGGAAACAGCTATGACTTTCCCTCATGTGCAGTGC) | KL11-150 (TGTAAAACGACGGCCAGTCACCTTTCATCCTTGCCCTA) | 533 | 1444-1976 |
| **4** | KL11-139 (CAGGAAACAGCTATGACTCTTGTGATAATCAACTGGATGC) | KL11-151 (TGTAAAACGACGGCCAGTCGAACTGTGCTACTGTTGAAGC) | 736 | 1757-2492 |
| **5** | KL11-140 (CAGGAAACAGCTATGACGGAAATAGCAGCGAATCGAA) | KL11-152 (TGTAAAACGACGGCCAGTCCAGTAACTCTACTATACCCAGTGCAT) | 1186 | 2123-3309 |
| **6** | KL11-141 (CAGGAAACAGCTATGACGGAGGTGTCTTCGTGGAATG) | KL11-153 (TGTAAAACGACGGCCAGTTGATGGCGCACCTCATAAT) | 1194 | 2629-3822 |
| **7** | KL11-142 (CAGGAAACAGCTATGACGCTAATATGGAATTTAACATGAAGGAA) | KL11-154 (TGTAAAACGACGGCCAGTGTGATTGGAGATGAGCCAAAG) | 779 | 3485-4263 |
| **8** | KL11-143 (CAGGAAACAGCTATGACCACATTTGGAGATTGGTGGA) | KL11-179 (TGTAAAACGACGGCCAGTGACTGGCTGCACTTTTCTACC) | 884 | 3922-4805 |
| **9** | KL11-174 (CAGGAAACAGCTATGACCGCCTGGTCGTGAAGTAGAG) | KL11-155 (TGTAAAACGACGGCCAGTAATTGTGTGCAACCCTTGAA) | 940 | 4440-5379 |
| **10** | KL11-144 (CAGGAAACAGCTATGACATGAGCGGAGGGGTTTCTAC) | KL11-180 (TGTAAAACGACGGCCAGTCCGTTTAAGCCTTGATGTCG) | 880 | 5012-5892 |
| **11** | KL11-175 (CAGGAAACAGCTATGACTTGATGGAAACTGTTCTGGATA) | KL11-156 (TGTAAAACGACGGCCAGTTTCCGTTTGAAAAAGTGTTGG) | 1079 | 5520-6598 |
| **12** | KL11-145 (CAGGAAACAGCTATGACAAGGTAAGGGCAAAGGCACT) | KL11-181 (TGTAAAACGACGGCCAGTCCATCATCAGTCGAAATCCA) | 883 | 6009-6891 |
| **13** | KL11-176 (CAGGAAACAGCTATGACCGAGTGTGCATGGTTAGCTC) | KL11-157 (TGTAAAACGACGGCCAGTTCCAACCCCCATAAAACTTG) | 1111 | 6767-7877 |
| **14** | KL11-146 (CAGGAAACAGCTATGACAGCTTGTGACAAAGCATGTTG) | KL11-158 (TGTAAAACGACGGCCAGTTTGGTTCTCGAGTCGAAGGT) | 1090 | 7254-8343 |
| **15** | KL11-147 (CAGGAAACAGCTATGACGCTCCCTGACACCATACTTGA) | KL11-149 (TGTAAAACGACGGCCAGTAGTGCCCTTCGTCCCAAC) | 986 | 7944-8929 |
| **16** | KL11-177 (CAGGAAACAGCTATGACAGCTTTACCCGAGCACAGAA) | KL11-148 (TGTAAAACGACGGCCAGTTCAAAAGCATAGCGAGCTAGA) | 955 | 8455-9409 |
| **17** | KL11-159 (CAGGAAACAGCTATGACACTCGAGCCACAATGCAAC) | KL04-33 (GGTCTCGAGTTTTTTTTTTTTTTT) | 1005 | 9053-10057 |

*M13 forward or reverse primer was used as adaptor to each viral specific primer to facilitate direct sequencing of the amplicons.
