# Supplementary material for: Deep Sequencing of Small RNAs in Tomato for Virus and Viroid Identification and Strain Differentiation
Source: PLoS One. 2012 May 18;7(5):e37127. doi: 10.1371/journal.pone.0037127 (PMC3356388; doi:10.1371/journal.pone.0037127)
Supplement: Table S2 — siRNA hot spot sequences and relative positions on Potato spindle tuber viroid in isolate CAHN8. (DOC) [file pone.0037127.s003.doc]

Table S2. The identified siRNA read counts and relative positions on *Potato spindle tuber viroid* in isolate CAHN8

|  | **sRNA** | **Size (nt)** | **Reads (order)** | **Sense (S) position** | **Antisense (AS) position** |
| --- | --- | --- | --- | --- | --- |
| HS1 | AGCGAACTGGCAATAAGGACGG | 22 | 4567 (3) | 107-128 (S) |  |
|  | GCGAACTGGCAATAAGGACGG | 21 | 8454 (1) | 108-128 (S) |  |
|  | GCGAACTGGCAATAAGGACGGT | 22 | 4744 (2) | 108-129 (S) |  |
| HS2 | CGAGTTTAGTTCCGAGGAACCA | 22 | 8021 (1) |  | 14-1, 358-351 (AS) |
|  | TTCCTCGGAACTAAACTCGTGG | 22 | 4648 (2) | 354-358,1-17 (S) |  |
|  | TCCTCGGAACTAAACTCGTGG | 21 | 3364 (3) | 355-358,1-17 (S) |  |
| HS3 | GAAAAAGCGGTTCTCGGGAGC | 21 | 6219 (3) |  | 300-280 (AS) |
|  | AGAAAAAGCGGTTCTCGGGAGC | 22 | 9364 (1) |  | 301-280 (AS) |
|  | AGAAAAAGCGGTTCTCGGGAG | 21 | 6384 (2) |  | 301-281 (AS) |
|  | AAGAAAAAGCGGTTCTCGGGAG | 22 | 5409 (4) |  | 302-281 (AS) |
|  | AAGAAAAAGCGGTTCTCGGGA | 21 | 5264 (5) |  | 302-282 (AS) |
