# Supplementary material for: Deep Sequencing of Small RNAs in Tomato for Virus and Viroid Identification and Strain Differentiation
Source: PLoS One. 2012 May 18;7(5):e37127. doi: 10.1371/journal.pone.0037127 (PMC3356388; doi:10.1371/journal.pone.0037127)
Supplement: Table S3 — Conserved siRNAs on the hotspots against Pepino mosaic virus EU strain in the three tomato samples. (DOC) [file pone.0037127.s004.doc]

Table S3. The conserved siRNA on the hotspots against *Pepino mosaic virus* EU strain in three tomato samples

| siRNA hot spot to PepMV-EU | Size (nt) | Reads  (order) | Reads  (order) | Reads (order) | Antisense (AS) position on PepMV-EU |
| --- | --- | --- | --- | --- | --- |
|  |  | CAHN8 | EF09_58 | EF09_60 |  |
| TGACTGTAGAATCAAGATGGTA | 22 | 213 (2) | 3520 (2) | 1164 (2) | 3990-3969 (AS) |
| TGACTGTAGAATCAAGATGGT | 21 | 395 (1) | 4225 (1) | 2003 (1) | 3990-3970 (AS) |
| TTGACTGTAGAATCAAGATGGT | 22 | 107 (3) | 1216 (3) | 530 (3) | 3991-3970 (AS) |
| TTGACTGTAGAATCAAGATGG | 21 | 62 (4) | 405 (4) | 229 (4) | 3991-3971 (AS) |
