# Supplementary material for: Deep Sequencing of Small RNAs in Tomato for Virus and Viroid Identification and Strain Differentiation
Source: PLoS One. 2012 May 18;7(5):e37127. doi: 10.1371/journal.pone.0037127 (PMC3356388; doi:10.1371/journal.pone.0037127)
Supplement: Table S4 — Conserved siRNA on the hotspots against Pepino mosaic virus US1 strain in the three independent samples. (DOC) [file pone.0037127.s005.doc]

**Table S4. The conserved siRNA on the hotspots against *Pepino mosaic virus* US1 strain in three independent samples**

| siRNA hot spot to PepMV-US1 | Size (nt) | Reads  (order) | Reads  (order) | Reads (order) | Antisense (AS) position on PepMV-US1 |
| --- | --- | --- | --- | --- | --- |
|  |  | CAHN8 | EF09_58 | EF09_60 |  |
| AGGAGATTGTCGACTAGCGGC | 21 | <50 | 361 (2) | 2888 (1) | 3472-3452 (AS) |
| CAGGAGATTGTCGACTAGCGGC | 22 | 171 (1) | 523 (1) | 1576 (2) | 3473-3452 (AS) |
| TCAGGAGATTGTCGACTAGCG | 21 | <50 | 109 (3) | 529 (3) | 3474-3454 (AS) |
| TCAGGAGATTGTCGACTAGCGG | 22 | <50 | 105 (4) | 351 (4) | 3474-3453 (AS) |
