# Supplementary material for: Deep Sequencing of Small RNAs in Tomato for Virus and Viroid Identification and Strain Differentiation
Source: PLoS One. 2012 May 18;7(5):e37127. doi: 10.1371/journal.pone.0037127 (PMC3356388; doi:10.1371/journal.pone.0037127)
Supplement: Table S5 — An unusual hot spot with siRNAs of upto 36 nt to PepMV-EU in the isolate EF09-58. (DOC) [file pone.0037127.s006.doc]

Table S5. An unusual hot spot with siRNA of 36 nt long to PepMV-EU in isolate EF09_58

| siRNA hot spot to PepMV-EU | Size (nt) | Reads | Sense position on PepMV-EU |
| --- | --- | --- | --- |
| CAAGAGGCAATCGGCAATTTACACACCGCTGCCAGA | 36 | 256 | 4200-4235 |
| AAGAGGCAATCGGCAATTTACACACCGCTGCCAGAT | 36 | 157 | 4201-4236 |
| AGAGGCAATCGGCAATTTACACACCGCTGCCAGATC | 36 | 255 | 4202-4237 |
| GAGGCAATCGGCAATTTACACACCGCTGCCAGATCG | 36 | 311 | 4203-4238 |
| AGGCAATCGGCAATTTACACACCGCTGCCAGATCGT | 36 | 249 | 4204-4239 |
| CAATCGGCAATTTACACACCGCTGCCAGA | 29 | 197 | 4207-4235 |
| AATCGGCAATTTACACACCGCTGCCAGA | 28 | 101 | 4208-4235 |
| ATCGGCAATTTACACACCGCTGCCAGA | 27 | 122 | 4209-4235 |
| TCGGCAATTTACACACCGCTGCCAGA | 26 | 161 | 4210-4235 |
